# Prognostic value of matrix metalloproteinase-9 expression in oral squamous cell carcinoma and its association with angiogenesis

**DOI:** 10.4317/jced.52712

**Published:** 2016-04-01

**Authors:** Azadeh Andisheh-Tadbir, Maryam Mardani, Sara Pourshahidi, Kamran Nezarati, Parisa Bahadori

**Affiliations:** 1 Associate Professor, Prevention of Oral and Dental Disease Research Center, School of Dentistry, Shiraz University of Medical Sciences, Shiraz, Iran; 2 Assistant Professor, Department of Oral Medicine, School of Dentistry, Shiraz University of Medical Sciences, Shiraz, Iran; 3 Assistant Professor, Department of Oral Medicine, School of Dentistry, Tehran University of Medical Sciences, Tehran, Iran; 4 Undergraduate Student, School of Dentistry, Shiraz University of Medical Sciences, Shiraz, Iran; 5 Undergraduate Student, School of Dentistry, International Branch, Shiraz University of Medical Sciences, Shiraz, Iran

## Abstract

**Background:**

Breakdown of extracellular matrix (ECM) is one of the important hallmarks of cancer progression which facilitates the invasion of tumoral cells to the surrounding tissue. Matrix metalloproteinases (MMPs) can degrade various components of the ECM and basement membrane. The aim of this study was to determine the role of matrix metalloproteinases-9 protein in the biologic behavior of oral squamous cell carcinoma (OSCC) and its relation with tumor angiogenesis.

**Material and Methods:**

In this study 42 OSCC and 15 normal epithelium were reviewed by immunohistochemical staining for matrix metalloproteinases-9 and CD105.

**Results:**

Matrix metalloproteinases-9 expression was detected in 32 OSCC specimens (76.1%), with 28 specimens (66.6%) showing moderate or strong expression. We observed that the expression level of matrix metalloproteinases-9 was positively correlated with the status of lymph node metastasis (N0vs. N1) (*P* =0.00), and clinical stage (I-II vs. III-IV) in OSCC patients. Microvessel density in intratumoral tissue has an association with lymph node metastasis and advanced clinical stage (*P*=0.003 and *p*=0.01, respectively). We observed that tumors with matrix metalloproteinases-9 overexpression had a higher microvessel density counts compared with tumors with absent or focal immunostaining(16.2±5.6 vs 10.3±3.5 respectively, *P* =0.03).

**Conclusions:**

In conclusion present results demonstrate the marked expression of matrix metalloproteinases-9 and CD105 in OSCC and suggest that the expression of these markers is associated with tumor progression and could offer additional information about the aggressiveness of OSCC. In addition a significant relationship was noted between microvessel density count and expression of matrix metalloproteinases-9 which suggest that MMP9 expression may be closely related to tumor angiogenesis.

** Key words:**Matrix metalloproteinases-9, CD105, squamous cell carcinoma, immunohistochemistry.

## Introduction

Squamous cell carcinoma (SCC) accounts for approximately 95% of oral malignant neoplasms and 38% of all malignant head and neck tumors (1). Different factors such as the degree of tumor differentiation, the proliferative activity of the tumor, and the invasion and metastatic potential affect the prognosis of SCC (2). Invasion and metastasis are multi-step processes that include basement membrane and ECM degradation, changes in cell adhesiveness, motility of tumor cells, and angiogenesis (3).

The investigation of the factors impacting these processes is important to understand tumor behavior and for the development of anticancer therapies.

Angiogenesis, which is critical for growth of tumor and metastasis, depends on the angiogenic factors which are produced by normal and tumoral cells. It implicates several pathways comprising the production of angiogenic factors, endothelial cells activation, and destruction of capillary membrane and migration of endothelial cells (4).

Increased vascularity improves the growth of primary neoplasms and increases the chance for hematogenous metastasis (5).

Microvessel density (MVD) is a quantitative method for analysis of angiogenesis which can be investigated by using various molecules, such as CD31, CD34, and CD105 (6,7).

CD105 is a homodimeric cell membrane glycoprotein and is a component of TGF-β receptor complex. This marker is an indicator of endothelial cell proliferation and is up-regulated during angiogenesis (8). In addition, expression of CD105 is one of the prominent characteristics of newly formed blood vessels and its expression is negative or insignificant in previously formed blood vessels, endothelium of the vessels of normal tissues and endothelial cells of lymphatic vessels (9).

Breakdown of ECM is one of the important hallmarks of cancer progression which facilitates the invasion of tumoral cells to the surrounding tissue. Matrix metalloproteinases (MMPs) are zinc dependent endopeptidases that can degrade various components of the ECM and basement membrane (BM) (10).

To date, at least 24 different MMP genes have been recognized in humans. MMPs are classified according to their substrate specificities collagenenases, gelatinases, stromelysins and matrilysins (11). Two different soluble gelatinase have been identified: gelatinase A, 72 KDa (MMP-2), and gelatinase B, 92KDa (MMP9) (12).

MMP9 has an important role in breakdown of ECM in normal physiological processes, such as embryonic development and tissue remodeling, as well as in the pathologic processes, such as a tumor metastasis (13).

MMP9 is secreted in a latent form and once activated, is able to degrade collagen in the ECM, which increases the metastasis of tumor cells (14).

The aim of the present study was to evaluate the immunohistochemical expression of MMP9 and CD105 in OSCC in order to determine the presence or absence of a correlation between the expression of these proteins and the clinicopathologic features.

## Material and Methods

-Materials

In this cross-sectional study, the specimen from 42 patients with oral SCC (28 males and 14 females) with a mean age 54.47 (range 35-81) from the archives of Pathology Department of Shiraz University of Medical Sciences (2008-2012) were studied. The control group was consisted of 15 cases of normal oral epithelium. This study was approved by the Ethics Committee of the Shiraz University of Medical Sciences.

-IHC staining and analysis

Firstly, Hematoxillin and Eosin slides of available blocks were reviewed and then cases with definite diagnosis and adequate cellular tissue were selected for immunohistochemical staining (IHC). IHC staining was performed by using EnvsionLabled Peroxides System (DAKO, Carpentaria, CA, USA). All the samples were fixed in 10% buffered formalin and were embedded in paraffin. Sections with 4μ-thickness were prepared, deparaffinized in xylene, rehydrated in graded alcohol and were washed with distilled water. Antigen retrieval was performed by using DAKO cytomation target retrieval solution with PH = 9, for 20 minutes. Internal peroxidase activity was inhibited by 3% H2O2.

Tissue sections were then incubated for 30 minutes with the anti-CD105 monoclonal antibody (mouse, Dako Corporation, Denmark) at a 1/10 dilution and with the anti-MMP-9 antibody (Santa Cruz Biotechnology Inc., Sc-19993) at a 1/50 dilution. Brown cytoplasmic staining for CD105 and MMP- 9 was considered as positive. Omission of primary antibody was considered as negative control, while liver tissue was used as positive control for CD105 and human colon adenocarcinoma for MMP-9.

For determining MVD hot-spot areas for CD105 expression in discrete blood vessels were initially identified by scanning the entire tumor of low power (X40) and the number of CD105 highlighted vessels in 10 of these areas was then counted in high power magnification (X400).

For analysis MMP-9 expression five microscopic fields in tumour tissues (original magnification 400×) were randomly selected and the percentage of positive cells was calculated. The percentage of positive cells was divided into four groups as follows: 0-25%,negative expression; 25-50%, weak expression; 50-75%,moderate expression; and 75-100%, strong expression. For statistical analysis, the patients were classified into two groups: ‘low expression’ included those with negative or weak expression and ‘high expression’ included those with moderate or strong expression.

-Statistical analysis

T-test, Independent samples t- test, paired T-test, Pearson correlation, and chi square test were used to compare the results between the two groups and the relation with clinicopathologic features. We used software SPSS15 to statically analyze the data. A *P*-value < 0.05 was considered significant in all the statistical analyses.

## Results

The clinicopathologic features of the patients included in this study are shown in  Table 1. The mean age of the investigated patients was 54.4±12.44 (range 35-81). Gender of the patients with OSCC included 28 males (66.7%) and 14 females (33.3%).

**Table 1:**
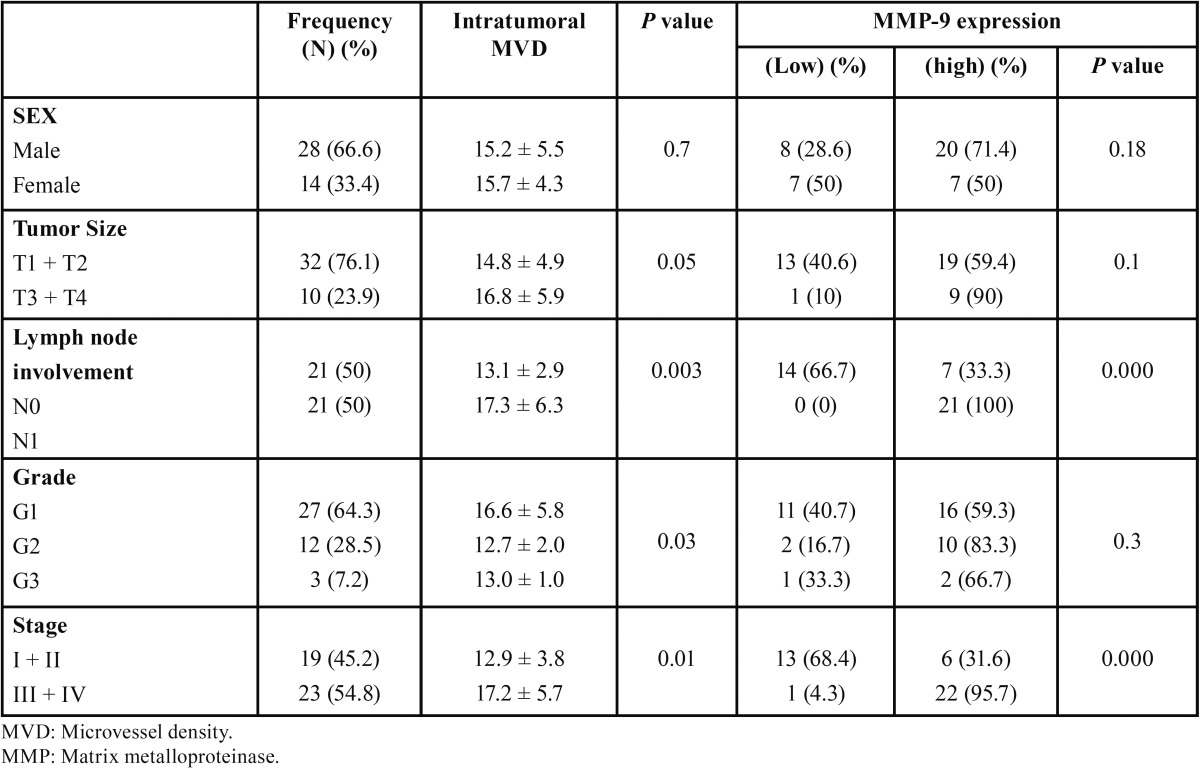
Relationship between MMP-9 expression, MVD and clinicopathologic parameters in patients with oral squamous cell carcinoma.

-Immunohistochemical analysis of MMP-9 expression

**Figure 1:**
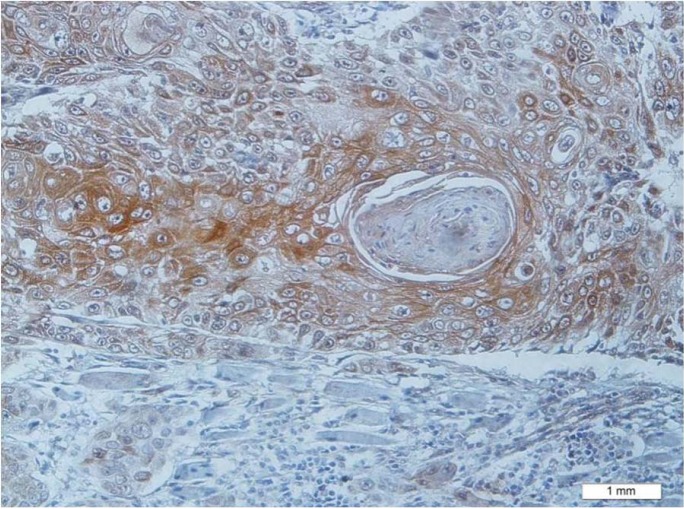
Diffuse expression of MMP-9 in tumoral cells of squamous cell carcinoma (×200).

**Figure 2:**
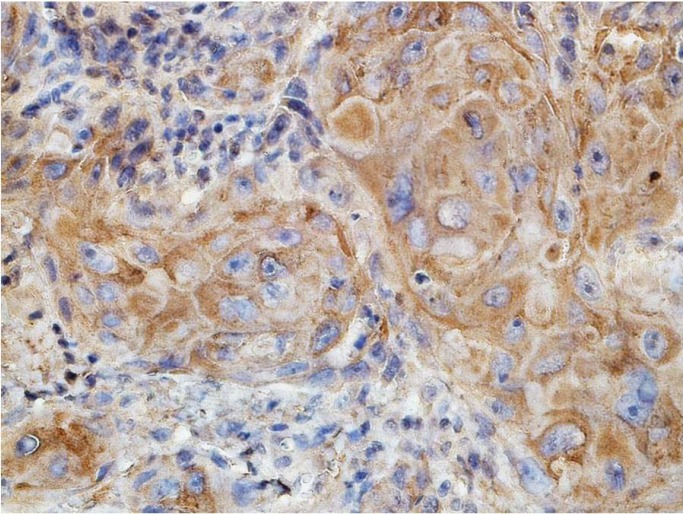
Diffuse cytoplasmic expression of MMP-9 in tumoral and stromal cells of squamous cell carcinoma (×400).

Positive expression of MMP-9 was mainly observed in the cytoplasm of stromal cells and proliferating epithelial cells as brownish granules under 400× (Figs. 1,2).

The expression of MMP-9 was not seen in normal oral epithelium. MMP-9 expression was detected in 32 OSCC specimens (76.1%), with 28 specimens (66.6%) showing moderate or strong expression.

-Relationship between clinicopathological characteristics and MMP9 expression in OSCC patients

The relationships between clinicopathological characteristics and MMP9 expression levels in individuals with OSCC are summarized in  Table 1. We did not find a significant association of MMP9 expression levels with patient’s age, sex, and tumor size (T classification). However, we observed that the expression level of MMP9 was positively correlated with the status of lymph node metastasis (N classification) (N0 vs. N1) (*P* =0.00), and clinical stage (I-II vs. III-IV) (*P* = 0.00) in OSCC patients ( Table 1).

-MVD assessment

**Figure 3:**
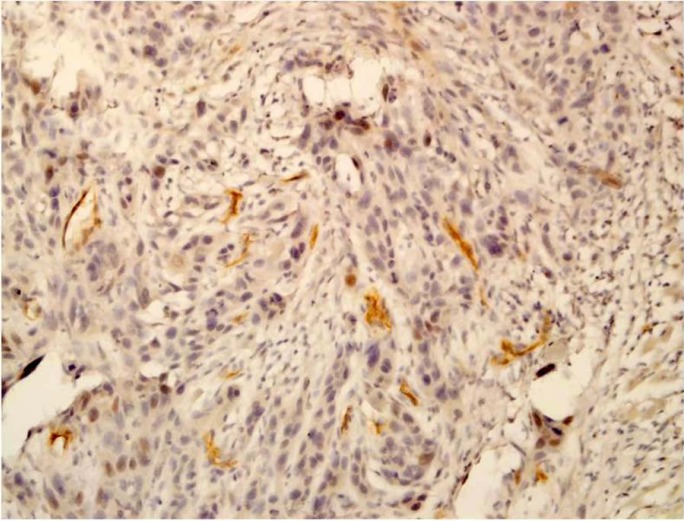
CD105 positive vessels in the stroma of squamous cell carcinoma (×200).

The mean CD105-MVD value was significantly higher in tumoral tissue (20.02±8.03) when compared to normal tissues (8.67±1.75) (Fig. 3). MVD in intratumoral tissue had an association with lymph node metastasis and advanced clinical stage (*P*=0.003 and *p*=0.01, respectively). There was no relation between the CD105-MVD value and tumor size in intratumoral area (*P*-value=0.05). Statistical analysis showed a negative association between grade of the tumor in intratumoral region (*P*-value=0.03). We did not observe any correlation between CD105-MVD with age and sex (*P*=0.4 and *p*=0.7 respectively). The correlation of the CD105-MVD value with clinicopathologic data is shown in  Table 1.

We analyzed the correlation between MVD and MMP9 expression and we observed that tumors with MMP-9 overexpression had a higher MVD counts compared with tumors with absent or focal immunostaining (16.2±5.6 vs 10.3±3.5, respectively *P* =0.03).

## Discussion

The expression and activation of proteolytic enzymes, which are involved in the ECM breakdown, is necessary for the invasion and spread of tumoral cells to the distant sites (15).

MMPs are a family of proteolytic enzymes with the ability of ECM degradation that has a critical role in several stages of tumor progression, consisting invasion, angiogenesis, and metastasis (16).

Among the various techniques that can be used to evaluate MMP expression in the tumoral tissue, immunohistochemistry demonstrates advantages; first because it permits direct correlation with morphology; and, second it can be used on paraffin embedded tissue. Therefore, it is useful for routine assessment of MMPs in diagnostic practice. Moreover, the immunohistochemicals studies are crucial since mRNA may not be translated, while protein expression has more functional relevance (17).

However, it has been proposed that immunohistochemistry cannot separate latent MMPs and active forms, while other techniques such as zymography can distinguish between the latent and active morphology (18).

Concerning MMP9, in the present study we found that 76.1% of OSCCs exhibited MMP9 immunoreactivity in tumor tissues and no expression is seen in normal tissues. These findings suggest that this molecule play an effective role in the development of these tumors.

In our study, the expression of MMP9 was found largely in tumor cells but also in the adjacent stromal and inflammatory cells. In view of this finding, our study supports previous findings and provides further evidence that both tumoral and stromal cells can produce different members of MMPs in tumor tissues. Indeed, it is possible that dynamic host-tumor interactions regulate MMPs level and affect the progression of human tumors (15).

Several studies demonstrated a relationship between the MMP9 expression and tumor aggressiveness in OSCC a fact that made this factor as a valuable marker for prognostic and therapeutic purposes (19). Werner *et al.* (20) showed that the overexpression of MMP9 is correlated with an invasive phenotype and metastatic potential of tumor cells.

In this study, MMP9 overexpression was significantly correlated with lymph node metastasis and advanced clinical stage. This finding reveals the significance of this enzyme in the aggressive phenotype of OSCCs through the destruction of ECM components, especially collagen IV (21).

Angiogenesis has been proposed to have a prognostic factor in tumors because it is necessary for tumor growth (5). In the current study we analyzed specimens from 42 patients diagnosed with OSCC and 15 normal specimens as the control group. Our study proved that CD105MVD was significantly higher in tumoral tissue compared with normal tissue which was in line with previous studies (22,23).

These results have verified that CD105 is more expressed in tumor tissues and may have a major role in the tumor. We also observed a positive relation between CD105 expression and lymph node metastasis. This finding was compatible with previous investigations (24-26) and suggested that the marker could be helpful in predicting the possibility of metastasis.

Angiogenesis is prerequisite for the growth, proliferation and metastasis of cancer cells. Therefore, these processes can be inhibited through inhibiting angiogenesis (27).

At present there are several antiangiogenic agents that affect the neovascularization pathway, but none of them has a predictive marker (28). High MVD alone is not a predictive marker for anti-angiogenic agents since anti-angiogenic agents target new vessels in tumors only. As CD105 expression is more specific for areas of neovascularization, CD105 has the potential to be a predictive marker for anti-angiogenic agents (27).

MMPs can improve tumor progression through affecting angiogenesis, because these enzymes degrade the ECM and offer a permissive microenvironment for the growth of new blood vessels. In this study, we observed that OSCC tumors with MMP9 overexpression had significantly higher MVD. Similar results were obtained previously by Franchi *et al.* (15) and Riedel *et al.* (29) in head and neck SCCs. A weak association also was found between MMP9 expression and tumor angiogenesis in breast carcinoma (30). Thus, MMP9 may be an important modulator of angiogenesis in OSCC, but further investigation is needed to clarify its specific mechanism of action.

In conclusion present results demonstrate the marked expression of MMP9 and CD105 in OSCC and suggest that the expression of these markers is associated with tumor progression and could offer additional information about the aggressiveness of OSCC. In addition a significant relationship was noted between MVD count and expression of MMP9 which suggest that MMP9 expression may be closely related to tumor angiogenesis.
